# Depression and post-traumatic stress disorder after perinatal loss in fathers: A systematic review

**DOI:** 10.1192/j.eurpsy.2022.2326

**Published:** 2022-10-28

**Authors:** Lieselotte Lamon, Marc De Hert, Johan Detraux, Titia Hompes

**Affiliations:** 1 University Psychiatric Centre, Katholieke Universiteit Leuven, Leuven, Belgium; 2 Department of Neurosciences, Centre for Clinical Psychiatry, Katholieke Universiteit Leuven, Leuven, Belgium; 3 Leuven Brain Institute, Katholieke Universiteit Leuven, Leuven, Belgium; 4 Antwerp Health Law and Ethics Chair, University of Antwerp, Antwerp, Belgium; 5 Department of Neurosciences, Public Health Psychiatry, Katholieke Universiteit Leuven, Leuven, Belgium; 6 Department of Neurosciences, Mind-Body Research, Katholieke Universiteit Leuven, Leuven, Belgium

**Keywords:** Depression, fathers, perinatal loss, PTSD

## Abstract

**Background:**

Research indicates that perinatal loss can cause profound psychological consequences in parents. However, a comprehensive summary of existing quantitative literature describing the association between perinatal loss and the development of depression/depressive symptoms or post-traumatic stress disorder (PTSD)/post-traumatic stress (PTS) symptoms in fathers has not been published.

**Methods:**

A systematic literature search (from inception to December 2021), using the PubMed, EMBASE, and Web of Science databases to articles assessing depressive or PTS symptoms, was conducted following the Preferred Reporting Items for Systematic reviews and Meta-Analyses guidelines. Only studies investigating the period of intrauterine death from 20 weeks of gestation, stillbirth, or neonatal death within the first month after birth were included.

**Results:**

A final sample of 13 articles were eligible for inclusion. Some studies showed an increased risk of depressive and PTS symptoms in fathers after perinatal loss. However, many study results did not show significant differences, symptoms generally decreased over time, and the majority of studies showed higher levels of depressive and PTS symptoms in mothers, compared with fathers.

**Conclusions:**

Although the majority of the included studies showed elevated levels of depressive and/or PTSD symptoms after perinatal loss in fathers, no clear firm conclusion can be drawn, as the included studies were very heterogeneous. More homogeneous research measuring depressive and PTS symptoms in fathers is needed at the time of the loss, as the current literature available shows several limitations and gaps.

## Background

**Figure 1.**
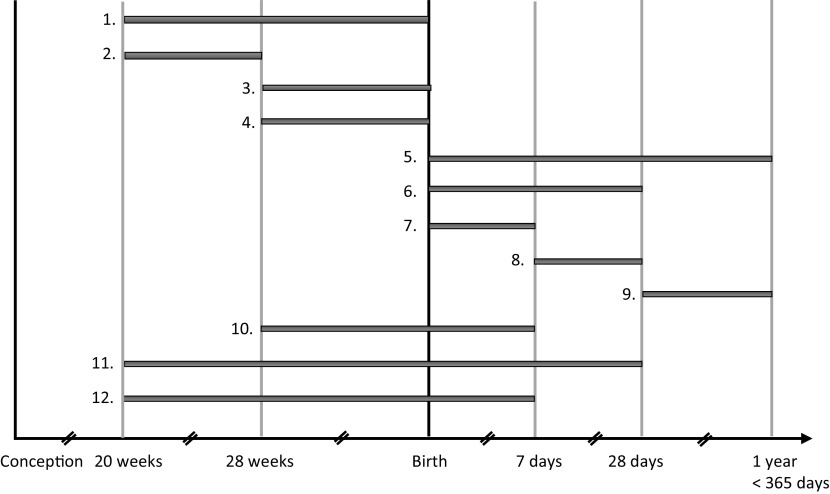
Standard terminology for fetal, infant, and perinatal deaths [1]. * is used when the definition is specifically used by World Health Organization (WHO) or National Center for Health Statistics (NCHS). 1) Stillbirth (Fetal deaths at 20 weeks’ gestation or more, fetus is defined from 8 weeks); 2) Early Fetal Death; 3) Late Fetal Death; 4) Stillbirth* (WHO); 5) Infant Death; 6) Neonatal Death; 7) Early Neonatal Death; 8) Late Neonatal Death; 9) Post Neonatal Death; 10) Perinatal Death* (WHO, NCHS); 11) Perinatal Death* (NCHS); 12) Perinatal Death.

Perinatal loss, defined in this systematic review as intrauterine death from 20 weeks of pregnancy or neonatal death within the first month after birth, is a trauma that many parents experience (see Figure 1 for an overview of different definitions of fetal, infant, and perinatal loss) [1]. According to the WHO, there are still approximately 2.6 million stillbirths each year [2, 3]. The WHO, likewise, states that in 2019 2.4 million newborns died in the first month of life, of which about 75% died in the first week [4]. It should be noted, however, that the prevalence of perinatal loss differs considerably in various parts of the world.

It is natural to experience psychological distress and grief reactions after perinatal loss: the loss of a child goes together with the loss of expectations, hopes, and dreams [5]. But in some cases, it can have more severe repercussions. Research indicates that there can be many psychological, physical, social, and financial consequences [6–8].

Perinatal loss has a major impact on both mothers and fathers. Only a few systematic reviews, however, showed that the grieving process and psychological consequences can differ significantly between men and women [9–12]. Fathers equally experience feelings of anxiety, depression, and post-traumatic stress (PTS) symptoms, but generally to a lesser extent than women [10, 12]. They show more avoidance or compensatory behavior like substance abuse and focus on work or specific tasks [7, 10, 11]. They often get the role to support their partner which can result in feelings of being overlooked and marginalized [6, 7, 10, 12]. Moreover, the risk of chronic grief symptoms or complicated grief in fathers is greater due to their tendency to suppress their feelings, hide their outward grief, and their difficulty in seeking help when needed [6, 7, 11]. It is therefore important that fathers also get social and community recognition for their grieving [9, 10, 12]. Furthermore, the incongruent grief and different coping mechanisms between parents can result in couple conflict with a great impact on the relationship [7].

Less is known about the explicit development of mental health conditions in parents, particularly in fathers. A recent systematic review and meta-analysis from Herbert et al. showed a significant association in women between perinatal loss and the development of mental health conditions like anxiety (Relative Risk (RR) = 1.75, 95% CI = 1.27–2.42, *p* < 0.001) and depression (RR = 2.14, 95% CI = 1.73–2.66, *p* < 0.001) [13]. The same systematic review did not show significant effects for the PTS outcomes [13]. Another recent systematic review of Westby et al. showed that in parents who experience stillbirth, the risk of reporting symptoms of depression or post-traumatic stress disorder (PTSD) is higher, compared to parents who did not experience stillbirth [14].

The purpose of this systematic review is to provide a comprehensive summary of existing quantitative literature that describes the association between perinatal loss experienced by fathers and depressive and/or stress symptoms. This provides added value to the current literature since the available literature on fathers’ experiences after perinatal loss is considerably less than that of mothers. Moreover, there is a social tendency after such loss to mainly take into account the mothers’ response. However, the potentially substantial impact perinatal loss can have on fathers should not be overlooked. The systematic review was conducted following the Preferred Reporting Items for Systematic Reviews and Meta-Analyses (PRISMA) guidelines [15].

## Methods

### Search strategy

A systematic literature search, using the PubMed, EMBASE, and Web of Science databases (from inception to December 2021), was conducted. Explorative searches were first performed to determine keywords. Search terms and full search strategies are available as Supplementary Material (see Supplement 1).

Duplicates were removed using Mendeley. The deduplicated results were imported into the internet-based program Rayyan for screening.

The first author (LL) performed the search strategy and screening process. In case of doubts about whether a reference should be included or not, the other authors (JD and MDH) independently reviewed the full text of the selected articles and assessed their eligibility.

### Eligibility criteria

Inclusion criteria were (a) peer-reviewed, original, and quantitative studies (cohort, cross-sectional and case–control studies); (b) studies published in English; (c) studies including fathers or couples; (d) studies in which fathers were assessed for depression/depressive symptoms or a PTSD/stress symptoms with at least one validated screening tool; (e) studies which described perinatal loss (women experiencing intrauterine death at 20 weeks of gestation or later, stillbirth or neonatal death within the first month after birth); and (f) controlled (including a control group of fathers with no experience of perinatal death) and non-controlled studies.

Reasons for exclusion were (a) studies in other languages than English; (b) studies for which no full text was available; (c) non-peer-reviewed articles (e.g., books, dissertations, conference abstracts); (d) qualitative studies, (systematic) reviews, and meta-analyses; (e) studies investigating the effect of specific interventions or social support on depression/depressive symptoms or PTSD/stress symptoms; (f) a study population without fathers; (g) studies not investigating the period of intrauterine death from 20 weeks of gestation, stillbirth, or neonatal death within the first month after birth; (h) studies without psychiatric outcome(s) (more specific the outcome depression/depressive symptoms or PTSD/stress symptoms); and (i) studies designed to test the effect of depression or stress disorders on perinatal adverse outcomes (see Table 1). Checking the reference lists of the included studies did not add any records meeting the inclusion criteria.

**Table 1.** In- and exclusion criteria.

Inclusion	Studies including fathers or couples Where women experienced intra-uterine death at 20 weeks of pregnancy or later, stillbirth, or neonatal death within the first 7 days after birth Fathers were assessed for depression/depressive symptoms or a stress disorder/symptoms with at least one validated screening tool Controlled (including a control group of fathers with no experience of perinatal death) and non-controlled studies Studies published in English or Dutch Quantitative observational studies: cohorts, cross sectional, and case–control studies Peer-reviewed articles
Exclusion	Studies in other languages than English or Dutch No full texts available Articles not peer reviewed like books, dissertations, or conference abstracts Qualitative studies, reviews, systematic reviews, and meta-analyses Studies investigating the effect of specific interventions or social support on depression or PTSD Study population without fathers Studies not investigating the period of intrauterine death from 20 weeks of gestation, stillbirth, or neonatal death within the first 7 days after birth Studies without psychiatric outcome(s): more specific the outcome depression/PTSD Studies designed to test the effect of depression or stress disorders on perinatal death

### Data extraction

Data from the included studies were extracted by LL to collect the following information: author, year, country, study design, time of loss, recruitment strategy, sample size, used validated scale to assess depression/depressive symptoms or PTSD/stress symptoms, number of assessments and time assessment points, and outcomes (depression/depressive symptoms or PTSD/stress symptoms).

### Quality assessment

One author (LL) assessed the quality of the included quantitative studies. The quality of each cohort study was rated using the Newcastle–Ottawa Scale (NOS) [16]. We modified the scale by adding a subdivision to the category for follow-up duration (follow-up duration longer than 6 months or a follow-up duration longer than 12 months). For the cohort studies, an overall score of 0–3 was considered low quality, 4–7 moderate quality, and 8–10 high quality. The quality of each cross-sectional study was rated using the adapted NOS [17], with a score of 0–3 indicating low quality, 4–7 moderate quality, and 8–10 high quality. Conflicting scores among the reviewers SL and JD were resolved by consensus and discussion.

## Results

### Search strategy

**Figure 2.**
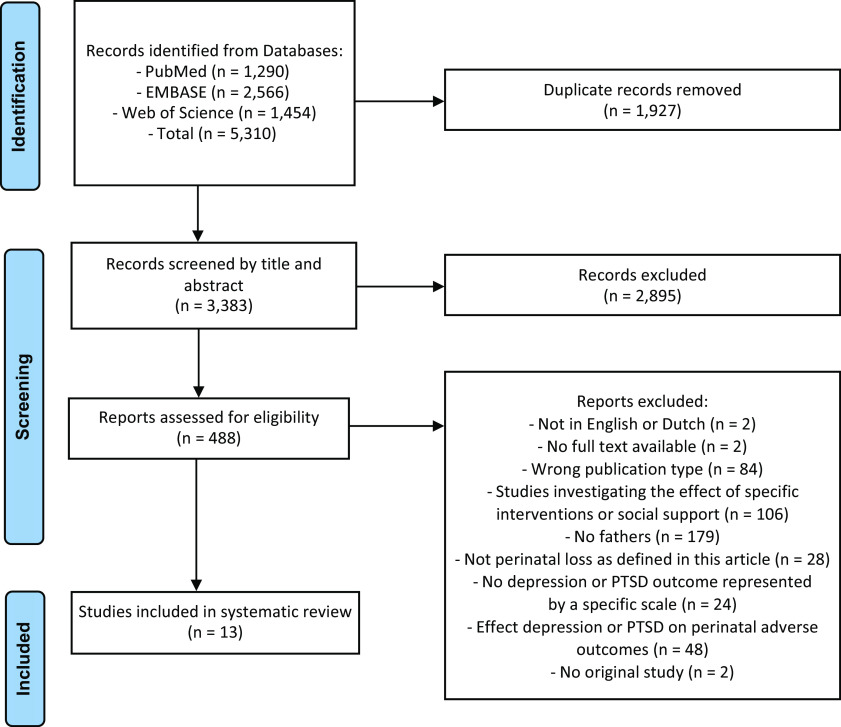
PRISMA flow diagram.

The original search in the PubMed (*n* = 1290), Embase (*n* = 2566), and Web of Science (*n* = 1454), databases yielded a total of 5310 reports. Of these, 1927 duplicate reports were removed. Overall, 488 articles were selected as potentially eligible, of which 13 original records met the inclusion criteria. Two articles of Christiansen et al. [18, 19] and two articles of Armstrong [20, 21] each were based on the same primary study. Checking the reference lists of the included studies did not add any records meeting the inclusion criteria. The results of the study selection are shown in the PRISMA flow diagram (see Figure 2).

### Description of the studies

The characteristics of the included original studies are shown in Table 2. Studies were published between 1988 and 2021. Overall, sample sizes were small, varying between 28 and 697 participants [25, 31]. Two studies exclusively included male participants [22, 25]. The time of loss went from miscarriage to infant death and most studies did not differentiate in results relating to the different times of loss. The number of assessments varied across studies from one to three. The time of assessment took place between 12 h and 20 years post-loss.

**Table 2.** Characteristics and outcomes of included studies

			Study characteristics					Psychiatric outcome	
References	Country	Study design	Time of perinatal loss	Recruitment strategy	Sample size (case/control)	Measures of depression and/or stress used	Numbers of assessment/time of assessment	Depression	PTSD
Riggs et al. [22]	Australia	Cross-sectional, survey study	PL from 4 to 28 weeks – SB > 20 weeks – Miscarriage <20 weeks	Participants (≥ 18 years) recruited via social media, stillbirth, and miscarriage support organizations and snowball sampling, had experienced a pregnancy loss no more recently than 1 year ago (so as to minimize potential distress)	48 participants (48 Australian cisgender heterosexual men/–)	DASS-21 (depression subscale)	One/time since the loss ranged from 1 to 20 years (mean: 4.2)	Prevalence of depression: On average participants often felt depressed (*p*-value not mentioned)	–
Sarkar et al. [23]	India	Cross-sectional study	SB > 28 weeks (or weighing 1000 g or more)	Study was conducted in a tertiary care public sector hospital Northern India between Mar 2019 and Jun 2020	600 participants (SB: 150 M and 150 F/LB: 150 M and 150 F)	EPDS-scale	One/postnatal visits 2–6 weeks after birth	Prevalence of depression in F: SB 18.1% vs LB 6.7%*	
Baransel and Uçar [24]	Turkey	Cross-sectional study	PL > 22 weeks (or from 500 grams) to the first 28 days after birth	Participants recruited from the obstetrics and newborn departments of a public hospital and a university hospital in the Malatya Province, between February and November 2017	308 participants (PL: 154 M and 154 F/–)	IES	One/12–24 hours post-loss	–	PTS symptoms n F: Concern for PTSD on the scale*** Gender effect:—PTS levels of M were higher than that of F *** – Subscales IES: Intrusion: higher in M, but between normal limits *** Avoidance: same in M and F, and between normal limits (0)
Roberts et al. [25]	India	Cross-sectional, survey study	SB (not specified)	Participants randomly recruited in a village where a mindfulness-based intervention was piloted for women who had a history of stillbirth, and healthcare providers who interact with fathers after stillbirth	28 participants (SB: 18 F/LB: 10 F)	HSCL-10	One/not specified	Depressive symptoms in F: higher levels SB group*	–
Murphy et al. [26]	UK	Cross-sectional, survey study	PL – Week 18 until birth Postnatal loss – Within the first 2 years of life	Members of the Danish National Association of Infant Death	565 participants (PL: 254 M and 191 F/LB: 64 M and 46 F)	TSC 35 (subscale depression)	One/time since the loss ranged from 5 days to 5 years (mean: 2.2 years)	Prevalence of depression (data consisting both M and F): – PL scored higher *** Gender effect: – M reporting higher levels of depressive symptoms than F***	–
Christiansen et al. [18, 19]	Denmark	Cross-sectional, survey study	ID prenatal: – 2^nd^ and 3^th^ trimester (42%) – > 22 weeks (92.3%) perinatal (11%) postnatal: up to 11 months (47%)	Participants (18–62 years) recruited from a private national support organization for parents bereaved by ID	634 participants (ID: 361 M and 273 F/–)	HTQ part IV	One/1.2 months to 18 years (mean: 3.4 years) post-loss	–	PTSD diagnosis: 12.3 % reached the criteria of probable PTSD diagnosis and 5.7% to subclinical PTSD ** Gender effect: – M higher PTSD severity and subscale scores * – Probable PTSD diagnosis: M almost twice as often as F (15.2% vs 8.4%) **
Armstrong et al. [27]	USA	A longitudinal cohort study	Prior PL fetal death (early or late) or neonatal death within the first 28 days of life	Participants (≥ 18 years) recruited from prenatal clinics, prenatal education classes, and private obstetric practices in a southeastern U.S. state, perinatal loss support groups and newsletters nationwide. Notices about the study were posted on Internet message boards focusing on PL as well as general pregnancy boards	72 participants (prior PL: 36 couples /–)	IES CES-D	Three/during and after pregnancy: prenatal (between 28 and 40 weeks) (T1); 2–3 months postpartum (T2); 6–8 months postpartum (T3)	Time effect (data consisting both M and F): – Depressive symptoms decreased as time proceeds *** Gender effect: – M more depressive symptoms than F at baseline, but not at T2 or T3**	Time effect (data consisting both M and F): – Overall PTS levels decreased * – Subscales IES: Intrusion increased ** Avoidance remained unchanged over the time (0) Gender effect: – Subscale effect: M higher intrusion *
Turton et al. [28]	UK	Community-based cohort study	Prior SB 2^nd^ trimester: 20–27 weeks (40.5%) 3th trimester: 28–41 weeks (59.5%)	Participants identified by screening consecutive case records in the antenatal clinics of three district general hospitals over a 3-year period	152 participants (prior SB: 38 couples/prior LB: 38 couples)	(T1) and (T2) – M EPDS – F BDI – M + F the PTSD–I Interview (T3)—M + F BDI the PTSD–I Interview	Three/after recent successful pregnancy: – 6 weeks (T1) – 6 months (T2) – 1 year (T3)	Levels of depression in F: Higher in the loss group (0) Time effect: Levels of depression falling as time proceeds * Gender effect: F symptoms were lower than those of M at all time points (0)	Levels of PTSD in F: Significant levels antenatally (15.6 % reached the criteria of current diagnosis PTSD and 18.8 % the criteria of lifetime diagnosis) Time effect: Symptoms remitted after a live birth (*p*-value not mentioned) Gender effect: – F symptoms were lower than those of M at all time points (0)
Armstrong [20, 21]	USA	Cross-sectional, survey study	Prior PL – 1^st^ trimester (29%) – 2^nd^ trimester (35%), – > 28 weeks (36%)	Participants (≥ 18 years) recruited from prenatal clinics, private obstetric practices, prenatal education classes, perinatal loss support groups, web sites, and newsletters	206 participants (prior PL: 40 couples/first pregnancy: 33 couples/prior LB: 30 couples	CES-D IES	One/between the 15^th^ and 32^nd^ week of the pregnancy	Depressive symptoms in F:—Higher level the PL group than in either of the 2 groups without loss (high risk of depression: loss group 23 % vs. first-time parents’ group 9%) * Gender effect:– M reporting higher levels of depressive symptoms than F **	Levels of stress in F: – High levels of continuing stress (90% F scored in the high stress range) Gender effect:—F 90% vs. M 88% scored in the high stress range (0) – Subscales IES M higher avoidance * F higher intrusion **
Franche and Mikail [29]	Canada	Cross-sectional, survey study	Prior PL – 1^st^ trimester (55%) – 2^nd^ trimester (22%), – 3^rd^ trimester (10%) – NDD (13%)	Participants recruited at the Obstetrics and Perinatology clinics of the Ottawa General Hospital, as well as at the clinics of obstetricians affiliated with the same hospital	113 participants (prior PL: 31 M and 28 F/prior LB: 31 M and 23 F)	BDI	One/between 10^th^ and 24^th^ week of the pregnancy	Depressive symptoms in F: Prior PL group scores higher on the scale (but still between normal limits) * Gender effect: M reporting higher levels of depressive symptoms than F ***	–
Franche and Bulow [30]	Canada	Cross-sectional, survey study	Prior PL and pregnancy – 10–41 weeks (mean: 25.24 weeks) (88% after 15 weeks) Prior PL and non-pregnancy– 1–42 weeks (mean: 27.72 weeks) (96% after 15 weeks)	Announcements concerning the study were posted in obstetrical clinics in a large Canadian university hospital. Recruitment for the pregnant loss group occurred between the 10^th^ and 19^th^ week of gestation, while recruitment for the non-pregnant loss group occurred at their 6^th^ week post-partum visit after prior PL	92 participants (prior PL and pregnancy: 25 M 24 F/prior PL and non-pregnancy: 25 M 18 F)	BDI	One/within 3 years after the PL during **pregnancy** or non-pregnancy	Depressive symptoms in F: Pregnant loss scores higher on the scale (but still between normal limits) (0) Gender effect: M reporting higher levels of depressive symptoms than F ***	–
Vance et al. [31]	UK	Cohort study	PL and SIDS SIDS, NND or SB combined (not specified)	Recruitment of 1 cohort of bereaved families who had experienced SIDS, NND, or SB. Attempts were made to contact all those who had lost babies in this way, between 1985 and 1988	697 participants (PL and SIDS: M 194 and F 143/LB: M 203 and F 157)	DSSI (depression subscale)	Four/2-, 8-, 15-, and 30-month post-loss	Levels of depression in F: PL 14.2 % vs. LB 2.7% at 2 months *** Gender effect: M reporting higher levels of depressive symptoms than F *	–
Theut et al. [32]	USA	Cross-sectional study	Prior PL – miscarriage (< 20 weeks) (64%) – SB (> 20 weeks) (28%) – NDD (8%)	Participants recruited via announcements in local newspapers, medical centers, and childbirth classes	112 participants (prior PL: 25 couples/first-time parents: 31 couples)	BDI	One/during the 8^th^ month of pregnancy	Depressive symptoms in F: – no difference between groups (all normal limits)	–

*Note:* M, mothers; F, fathers; SB, stillbirth; ID, infant death; PL, perinatal loss; NND, neonatal death; SIDS, sudden infant death syndrome; LB, live birth; PTS, post-traumatic stress; PTSD, post-traumatic stress disorder; T, time point; BDI, Beck Depression Inventory; CES-D, Center for Epidemiologic Studies Depression scale; DASS-21, The Depression, Anxiety and Stress Scale (21 Items); DSSI, Delusion Symptoms States Inventory; EPDS, Edinburgh Postnatal (Postpartum) Depression; IES, Impact of Event; HSCL-10, ten-item Hopkins Check List; HTQ, Harvard Trauma Questionnaire; PSS, Perceived Stress Scale; TSC 35, The Trauma Symptom Checklist (35 items).

Statistical significance is shown by * p <0.05, ** p<0.01, *** p<0.001. No statistical significance is shown by (0).

When assessment was taken in the subsequent pregnancy, the word pregnancy is highlighted in bold.

Most studies were conducted in high-income countries (HICs): three in the USA, four in Europe (one in Denmark and three in the UK), two in Canada, one in Turkey, and one in Australia. Two studies were conducted in India.

Six studies discussed prior perinatal loss, five had assessment points during a subsequent pregnancy, and one study discussed the difference in experience between couples who were expecting a child and couples who were not expecting a child, both after a previous perinatal loss.

### Study quality

For both cross-sectional and cohort studies, ratings of the quality assessment varied between four and eight. The quality of nine cross-sectional studies was considered moderate and the quality of one cross-sectional study was considered high. For the cohort studies, two studies had moderate quality and one study had high quality. Quality assessment of included studies is presented as Supplementary Material (see Supplement 2).

### Study results

Results are presented in two separate sections: depressive symptoms/depression and PTS symptoms/PTSD.

#### Depressive symptoms/depression

Eleven studies investigated depressive symptoms in parents after perinatal loss. Eight had a cross-sectional design and three a cohort design. One non-controlled cross-sectional survey study explored depressive symptoms exclusively in fathers. The scales that were used varied greatly. The DASS-21 (Depression, Anxiety and Stress Scale [21 Items]), HSCL-10 (ten-item Hopkins Symptom Check list), TSC 35 (Trauma Symptom Checklist [35 items]), and DSSI (Delusion Symptoms States Inventory) each were used in one study; the EPSD (Edinburgh Postnatal [Postpartum] Depression) scale was used in two studies, the CES-D (Center for Epidemiologic Studies Depression scale) in three studies, and the BDI (Beck Depression Inventory) in four studies (see Table 2).

A non-controlled study consisting of only male participants showed that fathers had a mean score for depression of 3.30 (SD = 0.87) on the DASS-21, which means that on average the fathers often felt depressed. But it was not mentioned if the results were statistically significant [22].

Furthermore, four studies compared the prevalence of depressive symptoms in parents experiencing a perinatal loss with those experiencing a live birth [23, 25, 26, 31]. The study by Murphy et al. described the long-term impact of pregnancy loss and infant death, measured 5 days to 5 years post-loss (with a mean of 2.2 years) on parents and showed a statistically significantly higher prevalence of depression in fathers of the loss group, compared to those experiencing a live birth (18.1% vs. 6.7%, *p* < 0.05) [26]. The study by Vance et al. showed 2 months after the loss a statistically significantly higher level of depression in fathers of the loss group, compared to those experiencing a live birth (14.2% vs. 2.7%, *p* < 0.001) [31]. Similarly, two other studies including a live birth control group showed statistically significantly elevated levels of depressive symptoms in fathers of the loss group, compared to those experiencing a live birth [23, 25].

Four other controlled studies described the prevalence of depressive symptoms during pregnancy after prior perinatal loss [20, 27, 29, 32]. Three of them found statistically significant elevated levels of depressive symptoms reported in the prior loss group, compared with the first-time fathers, or compared with fathers experiencing a prior live birth [20, 27, 29]. Franche and Mikail, however, did not observe a higher presence of depressive symptoms in fathers of the loss group, compared to those experiencing a live birth [29]. In contrast, in a study by Theut et al., which used a control group of first-time fathers, no statistically significant difference in depressive symptoms was found between fathers of the loss group and those experiencing a live birth [32].

Another study compared the effect of prior perinatal loss in pregnant and non-pregnant couples 3 years after the loss. The difference between fathers of pregnant and non-pregnant couples was not statistically significant, and the scores on the BDI remained within normal limits [30].

Turton et al. described the prevalence of depressive symptoms after a successful pregnancy between cases having experienced a previous loss and controls having had a previous live birth [28]. Although fathers of the previous loss group showed higher levels of depressive symptoms, the difference between both groups was not statistically significant [28].

Six studies described a gender effect in which mothers reported higher levels of depressive symptoms than fathers [21, 26, 27, 29–31]. Only in the study by Turton et al., the difference was not statistically significant [28].

Two longitudinal cohort studies described a statistically significant decline in depressive symptoms as time proceeds [27, 28].

#### *PTS symptoms*/*PTSD*

Five studies investigated PTS symptoms in parents after perinatal loss: three had a cross-sectional design and two a cohort design. Assessment scales varied greatly: HTQ (Harvard Trauma Questionnaire) part IV and the PTSD-I interview each were used in one study and the IES (Impact of Event Scale) in three studies.

Baransel and Uçar [24] found that the scores on the IES suggested a statistically significantly higher risk for PTSD 12 to 24 hours post-loss. Christiansen et al. found scores that statistically significantly correlated with a probable or subclinical diagnosis of PTSD in 12.3% and 5.7% of the fathers, respectively, 1.2 months to 18 years post-loss with a mean of 3.4 years (*p* < 0.01) [19]. A subsequent study, evaluating the gender effect, showed that mothers scored statistically significantly higher on PTSD severity and had a statistically significantly higher risk of probable PTSD diagnosis [18]. Another study by Armstrong described high levels of continuing stress in fathers during a subsequent pregnancy [21].

Furthermore, two studies described a time effect relating to PTS symptoms in fathers with prior loss after the birth of a subsequent child [27, 28]. Turton et al. found statistically significantly higher levels of PTSD in fathers antenatally: 15.6 % reached the criteria for current diagnosis PTSD and 18.8 % for a lifetime PTSD diagnosis. These symptoms, however, remitted completely after experiencing a live birth [28]. In accordance, Armstrong et al. found a statistically significant decrease in overall PTS symptoms over time after the birth of a subsequent child. However, it should be noted that these symptoms remained moderate in fathers even at 8 months postpartum. Moreover, intrusive thoughts statistically significantly increased, while avoidance of thoughts statistically significantly remained unchanged over time [27].

Finally, five studies investigated a gender effect [18, 21, 24, 27, 28]. Four of them described more overall PTS symptoms in mothers compared to fathers, although only in two studies, the difference was statistically significant [18, 24]. In a study by Armstrong, slightly more fathers (90%) scored within the high stress range, compared to mothers (88%), although this result was not statistically significant [21]. Three studies using the IES scale also reported results based on an intrusion and avoidance subscale. Although Baransel et al. showed statistically significantly higher intrusion scores in mothers than in fathers 12–24 hours post-loss, these remained within normal limits [24]. Likewise, Armstrong et al. found statistically significantly higher scores in mothers on the intrusion and avoidance scale [27]. An earlier study by Armstrong described higher scores on the avoidance scale in mothers but lower scores on the intrusion subscale [21]. In the latter two studies, fathers generally scored higher on the avoidance scale than on the intrusion scale [27].

## Discussion

Since the majority of the included studies showed elevated levels of depressive and PTS symptoms, and some studies specifically mention a higher prevalence of depression or PTSD, we can assume that fathers can be at a higher risk of developing depression or PTSD. Therefore, care workers should be attentive to the risk of developing these conditions, without pathologizing the grieving response of fathers as symptoms of grief and depressive/PTS symptoms can easily overlap.

Many different scales were used to measure depressive and PTS symptoms across studies. It should be noted that measurements of the response of parents after a perinatal loss in many studies were limited, meaning that important nuances could be missed. Moreover, the intensity and duration of responses can differ notably depending on different variables such as socio-economic status, cultural norms, social support, individual experiences, and relationships.

Another important note is the possibility to develop prolonged grief disorder (PGD), a condition recently introduced in DSM-5-TR [33]. As stipulated in the DSM-5 criteria, the symptoms of PGD cannot be explained by depression or PTSD. A recent meta-analysis stated that PGD is related to but can be distinguished from PTSD [34]. PGD after perinatal loss was not considered in our systematic review, as the concept was only introduced in the DSM-5-TR after this study was conducted. Yet, future research certainly should take this into account.

Nevertheless, it should be noted that the concept of PGD is still controversial and highly debated in the literature [35–41]. On the one hand, it should be considered that PGD can turn normal grieving responses into a mental health condition. This can lead to stigmatization and over-diagnosis with medicalization. On the other hand, it is suggested that diagnosing people with PGD after a loss can help them better understand what they are experiencing and get appropriate treatment. This interesting debate falls outside of the scope of our review. Moreover, the debate could be extended to the development of depression and/or PTSD after perinatal loss.

Some of the included studies were conducted at different periods, such as during a subsequent pregnancy or after the birth of a healthy child. Most studies found elevated levels of depressive symptoms during a subsequent pregnancy in fathers with prior perinatal loss, compared to fathers with prior live birth. Moreover, in general, PTS symptoms reached elevated levels [20, 21, 27, 29, 32]. These results correspond with previous literature [42]. Despite this, depressive and PTS symptoms generally decreased after the birth of a subsequent child [27, 28], suggesting a reduction of mental health conditions symptoms after a successful pregnancy. This conclusion, however, should be taken cautiously, as a higher level of anxiety about the well-being of the child has been described in parents during pregnancy and after the birth of a subsequent child [43]. Moreover, as the included studies were very heterogeneous no firm conclusion can be drawn.

Overall, in the included studies, mothers reported higher levels of symptoms than fathers. Only one study showed that fathers had slightly higher, but non-significant, scores within the high-stress range [21]. The gender difference was more pronounced for depressive compared to PTS symptoms.

There seems to be important gender differences in how fathers experience grief following perinatal loss. The father’s supportive role toward the mother, the father’s attachment to the baby during pregnancy, and women-focused maternity care and support services all may contribute to this gender difference [9, 44]. Moreover, more general variables may affect men’s responses such as traditional masculine societal norms [9, 44]. These traditional norms can influence their coping style and their decision to seek help [9, 44]. The gender role in the development and experience of depression or PTSD in men is acknowledged in the literature, and measures of depression and/or PTSD may not fully capture their response [45–47]. Notable, this gender role has mainly been researched in Western countries.

### Strengths and limitations

This systematic review has several strengths. We used a comprehensive and systematic research strategy with clearly defined in- and exclusion criteria. Both controlled and non-controlled studies were included, allowing more data to be included. The latter is relevant as most studies including a measurement of PTS symptoms did not include a control group. Another strength is that we only included studies using validated tools to identify depressive and/or PTS symptoms. Until now, the majority of systematic reviews on fathers experiencing a perinatal loss were only based on the reporting of subjective symptoms rather than scores on validated screening tools.

Despite this, several limitations need to be considered. Only one researcher (LL) systematically searched the databases for English language studies. However, doubts about in- or exclusion were discussed with the other authors. We also did not register our protocol prior to submitting the manuscript for publication. These limitations are present as this systematic review was conducted as a master’s thesis for obtaining a medical degree.

We defined perinatal loss as intrauterine death from 20 weeks of pregnancy or neonatal death within the first month after birth (see Figure 1) [1]. The terminology used for perinatal loss differs significantly across the literature, and the data of different types of perinatal loss were combined in many studies. This makes an adequate comparison of results difficult. Since one can expect different outcomes depending on the type of loss, it is important future research should be more consistent in describing the different types and timings of loss. This is important as previous research in mothers indicates an association between the length of gestational age with more grief and mental health condition symptoms [13, 48, 49].

In addition, losses like therapeutic termination of pregnancy (TtoP) were not included in the presented study. TtoP is an induced abortion after the second semester of gestation in the context of fetal unviability or causes of physical danger or pathological mental distress in the pregnant mother. Given the choice of undergoing a Ttop and the additional therapeutic effect, different effects on mental health conditions can be present [50].

Substantial variables that can influence the father’s experiences of the loss are not taken into account in this systematic review, such as the noteworthy effect of stigmatization and social support. In parents, experiences of stigmatization, rejection, discrimination, and overall silencing concerning the topic were reported [51, 52].

Finally, several limitations regarding the included studies were identified. First, studies often used different time assessment points (varying between hours to weeks, months and even years), making comparisons between study results difficult [19, 22, 26, 31]. Second, more studies were conducted in HICs (*n* = 11) than in low- to middle-income countries (LMICs, *n* = 2). Notably, less data are available in the literature about the effects of perinatal loss in LMIC, even though perinatal loss is more common [6]. In LMIC, there is a pronounced effect of cultural perspective on grief, care and social support are generally less extensive, and stigmatization about perinatal loss usually is more frequent [6, 7, 53]. Also, care workers have a different attitude toward the loss in LMIC and HIC [6]. These findings form a limitation to generalize the results across all countries and cultures. Third, various tools to identify depressive and/or PTS symptoms were used in the included studies. Sambrook Smith et al. suggest that for depressive symptoms EPDS, BDI, and PHQ are valid tools in diverse settings and perinatal populations. For PTS symptoms, there exist large gaps of evidence concerning the most valid tools [54]. Fourth, the included studies have population, sample, and response bias. The samples consisted of heterosexual couples. There are no data available on homosexual men or single fathers who experience perinatal loss together with women who plan to give the baby up for adoption. In addition, most studies on fathers consisted of studies that asked both members of the couple to participate. This can influence the results, as many of the included studies described an intercouple influence. Moreover, the sample mainly consisted of parents who volunteered to participate, automatically leading to positive bias towards parent who are, in advance, more interested in their mental health. Some included studies described recruitment difficulties of fathers who experienced perinatal loss, which can explain the small sample sizes.

### Implications for clinical practice and/or research

Fathers can also experience negative consequences of the perinatal loss, and this should not be overlooked. The present systematic review gives a better insight into whether fathers cross the threshold from natural grieving reactions to specific mental health conditions and allows healthcare workers, family, and friends to better assess their needs for appropriate care.

Fathers should get appropriate and tailored care, as depressive and PTS symptoms can have an important effect on their quality of life and their relationships. Overall, parents develop a positive relationship with their subsequent child [43]. However, some couples experience difficulties regarding attachment and bonding with their children and handle different parenting techniques [7, 55, 56]. In the light of the importance of the first 1000 days, covering the period between conception and the first 2 years after birth, early detection of mental health problems due to having experienced a previous birth loss in (expectant) mothers as well as fathers is fundamental. Moreover, a difference in the grieving process after perinatal loss between men and women may be present, and as a result, relationship issues like divorce, conflicting emotional reactions toward sexual relationships, and even spousal abuse can arise [6, 7, 57].

Therefore, men’s perinatal/neonatal loss should be equally recognized as the grief of their partners. Until present, little is known about specific interventions providing help to fathers [6], but evidence is growing that tailored and appropriate support options that suit men, using multilevel strategies, are needed [9]. The socio-ecological model of men’s grief implies that beyond individual and interpersonal supports, there is also a need to educate the community about the impact of pregnancy/neonatal loss on men, as well as promoting their strengths to seek and accept, rather than avoid [9].

It is paramount that healthcare providers involved in taking care of parents facing perinatal loss are trained in supporting them in their grief in a sensitive and empathic manner. They should be able to, at least, provide explanations to bereaved parents about incongruent grieving between partners, and skills to navigate potential issues. Appropriate, jargon-free language should be used, providing explanations relating to the cause of loss when available. Follow-up calls specifically to men in the weeks or months following a loss should be made. Furthermore, men pointed out that practical information on how best to support their partner, alongside recognizing and managing their own grief, is useful [58–60]

In addition, solid communication between healthcare providers, from all tires, surrounding these parents is important. This is in an effort to guide these parents, whenever necessary in the period after perinatal/neonatal loss but also during subsequent pregnancies. In this way, proper support can be given and chances to detect pathology in an early stage can be optimized.

## Conclusion

Although the majority of the included studies showed elevated levels of depressive and/or PTSD symptoms after perinatal loss in fathers, no clear firm conclusion can be drawn, as the included studies were very heterogeneous. Nevertheless, there are enough indications that perinatal loss may increase the risk of depressive and PTS symptoms in fathers. Therefore, sufficient attention should be given to fathers after experiencing perinatal loss.

## Data Availability

The analysis is based on the content of the selected publications.

## Supplementary material

For supplementary material accompanying this paper visit https://doi.org/10.1192/j.eurpsy.2022.2326. click here to view supplementary material
