# Supplementary material for: Depression and post-traumatic stress disorder after perinatal loss in fathers: A systematic review
Source: Eur Psychiatry. 2022 Oct 28;65(1):e72. doi: 10.1192/j.eurpsy.2022.2326 (PMC9677450; doi:10.1192/j.eurpsy.2022.2326)
Supplement: Supplementary file 1 [file S0924933822023264sup001.docx]

**Supplement 1. Search terms.**

**Search terms**

| fetal mortalit* foetal mortalit*  fetus mortalit*  foetus mortalit*  fetal death*  fetus death*  foetus death*  death fetus  death foetus  dead fetus  dead foetus  foetal death*  fetal demise  foetal demise  stillbirth*  still birth*  stillborn  still born | perinatal death*  prenatal death*  infant mortalit*  infantile mortalit*  infant death*  perinatal loss*  perinatal mortalit*  neonatal death*  neonate death*  newborn death*  new born death*  newborn mortalit*  new born mortalit*  baby death*  pregnancy loss*  neonatal loss*  neonatal mortalit* | intrauterine death*  intra uterine death*  intrauterine fetal death*  intra uterine fetal death*  intrauterine foetal death*  intra uterine foetal death*  IUFD  intrauterine loss*  intra uterine loss*  antepartum death*  endouterine death*  postnatal death*  postnatal mortalit*  postnatal loss* |
| --- | --- | --- |
| depress*  melanchol* | dysthymi*  disthymi* | MDD  dysthimi* |
| posttraumatic neuros*  post-traumatic neuros*  posttraumatic stress*  post-traumatic stress*  posttraumatic syndrome* | post-traumatic syndrome*  PTSD  Trauma and stressor related disorder*  traumatic stress* | acute stress disorder*  acute traumatic stress disorder*  acute stress reaction  reaction to severe stress  adjustment disorder* |

**Search strategy**

| *PUBMED (1,290 results)*  ("Fetal Mortality"[Mesh] OR "fetal mortalit*"[tiab] OR "foetal mortalit*"[tiab] OR "fetus mortalit*"[tiab] OR "foetus mortalit*"[tiab] OR "Fetal Death"[Mesh] OR "fetal death*"[tiab] OR "fetus death*"[tiab] OR "foetus death*"[tiab] OR " death fetus"[tiab] OR "death foetus"[tiab] OR "dead fetus"[tiab] OR "dead foetus"[tiab]OR "foetal death*"[tiab] OR "antepartum death*"[tiab] OR "endouterine death*"[tiab] OR "fetal demise"[tiab] OR "foetal demise"[tiab] OR "stillbirth*"[tiab] OR "still birth*"[tiab] OR "stillborn"[tiab] OR "still born"[tiab] OR "Perinatal Death"[Mesh] OR "perinatal death*"[tiab] OR "prenatal death*"[tiab] OR "Infant Mortality"[Mesh] OR "infant mortalit*"[tiab] OR "infantile mortalit*"[tiab] OR "Infant Death"[Mesh] OR "infant death*"[tiab] OR "perinatal loss*"[tiab] OR "Perinatal Mortality"[Mesh] OR "perinatal mortalit*"[tiab] OR "neonatal death*"[tiab] OR "neonate death*"[tiab] OR "newborn death*"[tiab] OR "new born death*"[tiab] OR "newborn mortalit*"[tiab] OR "new born mortalit*"[tiab] OR "baby death*"[tiab] OR "pregnancy loss*"[tiab] OR "neonatal loss*"[tiab] OR "neonatal mortalit*"[tiab] OR "intrauterine death*"[tiab] OR "intra uterine death*"[tiab] OR "intrauterine fetal death*"[tiab] OR "intra uterine fetal death*"[tiab] OR "intrauterine foetal death*"[tiab] OR "intra uterine foetal death*"[tiab] OR "IUFD"[tiab] OR "intrauterine loss*"[tiab] OR "intra uterine loss*"[tiab] OR "postnatal death*"[tiab] OR "postnatal mortalit*"[tiab] OR "postnatal loss*"[tiab]) AND ("Depression"[Mesh] OR "Depressive Disorder"[Mesh] OR "depress*"[tiab] OR "melanchol*"[tiab] OR "dysthymi*"[tiab] OR "disthymi*"[tiab] OR "dysthimi*"[tiab] OR “MDD”[tiab] OR "Trauma and Stressor Related Disorders"[Mesh] OR "trauma and stressor related disorder*"[tiab] OR "posttraumatic neuros*"[tiab] OR "post-traumatic neuros*"[tiab] OR "posttraumatic stress*"[tiab] OR "post-traumatic stress*"[tiab] OR "PTSD"[tiab] OR "posttraumatic syndrome*"[tiab] OR "post-traumatic syndrome*"[tiab] OR "acute stress disorder*"[tiab] OR "acute traumatic stress disorder*"[tiab] OR "acute stress reaction"[tiab] OR "reaction to severe stress"[tiab] OR "adjustment disorder*"[tiab] OR “traumatic stress*”[tiab]) |
| --- |
| *EMBASE (2,566 results)*  (‘fetus mortality’/exp OR ‘fetus mortalit*’:ti,ab,kw OR ‘fetus death’/exp OR ‘fetus death*’:ti,ab,kw OR ‘foetus death*’:ti,ab,kw OR ‘death fetus ’:ti,ab,kw OR ‘death foetus ’:ti,ab,kw OR ‘dead fetus ’:ti,ab,kw OR ‘dead foetus ’:ti,ab,kw OR ‘fetal mortalit*’:ti,ab,kw OR ‘foetal mortalit*’:ti,ab,kw OR ‘foetus mortalit*’:ti,ab,kw OR ‘fetal death*’:ti,ab,kw OR ‘foetal death*’:ti,ab,kw OR ‘antepartum death*’:ti,ab,kw OR ‘endouterine death*’:ti,ab,kw OR ‘fetal demise’:ti,ab,kw OR ‘foetal demise’:ti,ab,kw OR ‘stillbirth*’:ti,ab,kw OR ‘stillborn’:ti,ab,kw OR ‘still birth*’:ti,ab,kw OR ‘still born’:ti,ab,kw OR ‘perinatal death’/exp OR ‘perinatal mortality’/exp OR ‘perinatal death*’:ti,ab,kw OR ‘prenatal death*’:ti,ab,kw OR ‘perinatal loss*’:ti,ab,kw OR ‘perinatal mortalit*’:ti,ab,kw OR ‘infant mortality’/exp OR ‘infant mortalit*’:ti,ab,kw OR ‘infantile mortalit*’:ti,ab,kw OR ‘neonatal death*’:ti,ab,kw OR ‘neonate death*’:ti,ab,kw OR ‘neonatal loss*’:ti,ab,kw OR ‘neonatal mortalit*’:ti,ab,kw OR ‘intrauterine death’:ti,ab,kw OR ‘intra uterine death’:ti,ab,kw OR ‘intra uterine fetal death’:ti,ab,kw OR ‘intrauterine fetal death’:ti,ab,kw OR ‘intra uterine foetal death’:ti,ab,kw OR ‘intrauterine foetal death’:ti,ab,kw OR ‘intrauterine loss’:ti,ab,kw OR ‘intra uterine loss’:ti,ab,kw OR ‘IUFD’:ti,ab,kw OR ‘postnatal death*’:ti,ab,kw OR ‘postnatal mortalit*’:ti,ab,kw OR ‘postnatal loss*’:ti,ab,kw OR ‘newborn death’/exp OR ‘newborn death*’:ti,ab,kw OR ‘new born death*’:ti,ab,kw OR ‘newborn mortalit*’:ti,ab,kw OR ‘new born mortalit*’:ti,ab,kw OR ‘baby death*’:ti,ab,kw OR ‘pregnancy loss’/exp OR ‘pregnancy loss*’:ti,ab,kw OR ‘infant death*’:ti,ab,kw) AND (‘depression’/exp OR ‘depress*’:ti,ab,kw OR ‘melanchol*’:ti,ab,kw OR ‘dysthymi*’:ti,ab,kw OR ‘disthymi*’:ti,ab,kw OR ‘dysthimi*’:ti,ab,kw OR ‘MDD’:ti,ab,kw OR ‘posttraumatic stress disorder’/exp OR ‘trauma and stressor related disorder*’:ti,ab,kw OR ‘acute stress disorder’/exp OR ‘adjustment disorder’/exp OR ‘posttraumatic neuros*’:ti,ab,kw OR ‘post-traumatic neuros*’:ti,ab,kw OR ‘posttraumatic stress*’:ti,ab,kw OR ‘post-traumatic stress*’:ti,ab,kw OR ‘PTSD’:ti,ab,kw OR ‘posttraumatic syndrome*’:ti,ab,kw OR ‘post-traumatic syndrome*’:ti,ab,kw OR ‘acute stress disorder*’:ti,ab,kw OR ‘acute traumatic stress disorder*’:ti,ab,kw OR ‘acute stress reaction*’:ti,ab,kw OR ‘reaction to severe stress’:ti,ab,kw OR ‘adjustment disorder*’:ti,ab,kw OR ‘traumatic stress*’:ti,ab,kw) |
| *WEB OF SCIENCE (1,454 results)*  TS=("fetal mortalit*" OR "foetal mortalit*" OR "fetus mortalit*" OR "foetus mortalit*" OR "fetal death*" OR "fetus death*" OR "foetus death*" OR " death fetus" OR "death foetus" OR "dead fetus" OR "dead foetus" OR "foetal death*" OR "antepartum death*" OR "endouterine death*" OR "fetal demise" OR "foetal demise" OR "stillbirth*" OR "still birth*" OR "stillborn" OR "still born" OR "perinatal death*" OR "prenatal death*" OR "infant mortalit*" OR "infantile mortalit*" OR "infant death*" OR "perinatal loss*" OR "perinatal mortalit*" OR "neonatal death*" OR "neonate death*" OR "newborn death*" OR "new born death*" OR "newborn mortalit*" OR "new born mortalit*" OR "baby death*" OR "pregnancy loss*" OR "neonatal loss*" OR "neonatal mortalit*" OR "intrauterine death*" OR "intra uterine death*" OR "intrauterine fetal death*" OR "intra uterine fetal death*" OR "intrauterine foetal death*" OR "intra uterine foetal death*" OR "IUFD" OR "intrauterine loss*" OR "intra uterine loss*" OR "postnatal death*" OR "postnatal mortalit*" OR "postnatal loss*") AND TS=("depress*" OR "melanchol*" OR "dysthymi*" OR "disthymi*" OR "dysthimi*" OR “MDD” OR "trauma and stressor related disorder*"[tiab] OR "posttraumatic neuros*" OR "post-traumatic neuros*" OR "posttraumatic stress*" OR "post-traumatic stress*" OR "PTSD" OR "posttraumatic syndrome*"[tiab] OR "post-traumatic syndrome*"[tiab] OR "acute stress disorder*" OR "acute traumatic stress disorder*" OR "acute stress reaction" OR "reaction to severe stress" OR "adjustment disorder*" OR “traumatic stress*”) |

**Supplement 2. Quality assessment.**

**Quality assessment of cross-sectional studies included in the systematic review using the adapted Newcastle-Ottawa Scale (NOS)**

|  | Selection | Comparibility | Outcome | Total score  Maximum 10 |
| --- | --- | --- | --- | --- |
| Riggs et al. | **** | * | * | 6 |
| Sarkar et al. | **** | ** | * | 7 |
| Baransel et al. | **** | ** | ** | 8 |
| Roberts et al. | ** | ** | * | 5 |
| Murphy et al. | *** |  | * | 4 |
| Christiansen et al. | *** | * | ** | 6 |
| Armstrong | *** | * | ** | 6 |
| Franche & Mikail | *** | * | ** | 6 |
| Franche & Bulow | **** |  | ** | 6 |
| Theut et al. | *** |  | * | 4 |
|  | * Sample size > 25 fathers after perinatal loss | **Adjustment for the main confounding factors: perinatal loss characteristics and antenatal characteristics  *Adjustment for only one of the main confounding factors or for other confounding factors |  | 0-3 low; 4-7 moderate; 8-10 high quality |

**Quality assessment of cohort studies included in the systematic review using the Newcastle-Ottawa Scale (NOS)**

|  | Selection | Comparibility | Outcome | Total score  Maximum 10 |
| --- | --- | --- | --- | --- |
| Armstrong et al. | * | * | ** | 4 |
| Turton et al. | *** | ** | *** | 8 |
| Vance et al. | ** |  | ** | 4 |
|  |  | **Adjustment for the main confounding factors: perinatal loss characteristics and antenatal characteristics  *Adjustment for only one of the main confounding factors or for other confounding factors | ** Follow-up duration > 12 month * Follow-up duration > 6 months | 0-3 low; 4-7 moderate; 8-10 high quality |
